# Women’s experiences of receiving a diagnosis of premenstrual dysphoric disorder: a qualitative investigation

**DOI:** 10.1186/s12905-020-01100-8

**Published:** 2020-10-28

**Authors:** Elizabeth Osborn, Anja Wittkowski, Joanna Brooks, Paula E. Briggs, P. M. Shaughn O’Brien

**Affiliations:** 1 Division of Psychology and Mental Health, School of Health Sciences, Faculty of Biology, Medicine and Health, Manchester Academic Health Science Centre, The University of Manchester, 2nd Floor Zochonis Building, Brunswick Street, Manchester, M13 9PL UK; 2 Greater Manchester Mental Health NHS Foundation Trust, Manchester, UK; 3 Liverpool Women’s Hospital, Liverpool, UK; 4 Emeritus Professor, School of Medicine, Keele University, Staffordshire, UK

**Keywords:** PMDD, Qualitative methodology, Misdiagnosis, Women’s health

## Abstract

**Background:**

Premenstrual dysphoric disorder (PMDD) is a complex and disabling condition that affects women of reproductive age, characterised by severe physical and psychological symptoms that occur cyclically and remit following the onset of menses. As the psychological nature and consequences of PMDD often seem indistinguishable from symptoms of other mental health difficulties, this condition presents distinct diagnostic challenges for healthcare professionals. Therefore, this study aimed to explore women’s experiences of both having PMDD and of receiving this diagnosis.

**Methods:**

Participant recruitment took place in the United Kingdom during 2018. Seventeen women who had been diagnosed with PMDD by a medical specialist and met the clinical criteria for PMDD on the premenstrual symptoms screening tool were interviewed. The data from these semi-structured interviews were audio-recorded, transcribed and inductively analysed using reflexive thematic analysis.

**Results:**

Twelve subthemes were identified and organised around four main themes: (1) *A broken woman,* (2) *Misdiagnosis and the lost decades,* (3) *A life transformed* and (4) *Negotiating the aftermath.*

**Conclusions:**

The findings of this study highlight the critical importance of the accurate and timely detection of PMDD, with the aim of preventing women from experiencing severe and prolonged psychological distress. In order to achieve this, there needs to be a greater understanding and awareness of PMDD within both the medical and lay communities, alongside training for healthcare practitioners in PMDD assessment.

## Background

Premenstrual dysphoric disorder (PMDD) is a complex and disabling condition affecting women of reproductive age. Prior to the onset of menses, women with PMDD experience various cognitive, psychological and somatic symptoms that occur cyclically in response to normal hormonal changes [1]. Fluctuating levels of oestrogen and progesterone produce debilitating emotional and behavioural symptoms, including experiences of severe depression and anxiety [2] and even episodes of psychosis [3], which remit shortly after the onset of menstruation [1]. Left untreated, these symptoms can cause considerable impairment to women’s quality of life [4, 5] and have resulted in many women having attempted, and in some cases succeeded, in taking their own lives [6–8].

Prevalence estimates for PMDD range from between 3 and 8% of all menstruating women [4], yet there remains limited awareness and understanding of PMDD in the medical community, alongside conflicting views over its validity as a diagnostic category [9] which stem from differences in the way that the condition has historically been conceptualised. Having formerly been referred to as a severe form of Premenstrual Syndrome (PMS) and subsequently renamed and classified by American psychiatrists as a mental disorder [10, 11], PMDD has been a source of much controversy surrounding the ‘medicalisation of women’s bodies’ [12, 13]. Whilst PMDD is still currently included as a psychiatric condition within the DSM-5 [11], there have been a number of new developments that have challenged this position.

A recent proliferation of research has identified key neurobiological differences for women who present with this condition, including abnormal expressions of hormone-processing genes [14] and altered brain sensitivities to allopregnanolone, a main progesterone metabolite [15–17]. Whilst a definitive aetiology of PMDD remains unknown [18], these findings have helped to substantiate PMDD as a legitimate biological disease that requires both medical attention and intervention [1] and, in 2019, led the World Health Organisation to announce the inclusion of PMDD in the eleventh revision of the International Classification of Diseases and Related Health Problems (ICD-11) as a disease of the genitourinary system [19]. These different classifications of PMDD, as both a psychiatric condition within the DSM-5 and a medical condition within the ICD-11, illustrate the complexity of differentiating physical and mental health conditions. However, as healthcare systems are traditionally split into treating either medical or mental health difficulties, the latest understanding that “…PMDD is not a mental health condition to be treated by a psychiatrist—but a neuro-hormonal disorder that falls squarely into the gynaecological remit” [20] appears critical to ensuring that women with this condition are able to access the most appropriate treatments.

In the absence of a definitive biological marker for PMDD, diagnosis currently relies on the knowledge and expertise of treating healthcare professionals [21]. With the psychological consequences of PMDD often presenting as indistinguishable from symptoms of other mental health conditions, diagnosis is based on the cyclical timing and prospective charting of women’s symptoms in order to determine their relationship to the onset of menstruation [11, 22]. When PMDD is accurately diagnosed, there are treatment options available to manage symptoms, including ovulation suppression with a GnRH agonist and hysterectomy combined with bilateral oophorectomy as a last resort option [1, 23]. However, owing to the complexity of diagnosis and poor understanding of PMDD by most health professionals, women can experience many years of unrecognised and untreated symptoms, and mental health misdiagnoses [3, 5].

There are a number of qualitative studies investigating women’s views and experiences of PMS. However, as women with PMDD are now recognised to represent a distinct clinical entity and a much more severely afflicted category of women [19], it is important to further understand the experiences of this specific population. In one previous qualitative study 15 women with PMDD were interviewed about their workplace experiences. Using a thematic analysis approach, two main themes were generated pertaining to the different “phases” of PMDD that women experienced during their menstrual cycle and to a lack of awareness and support within organisations [24]. However, as this study was limited to reviewing PMDD in relation to the work context, we sought to further understand women’s broader experiences of having this condition. Therefore, the aim of this qualitative study was to explore women’s narratives of both having PMDD and of receiving this diagnosis.

## Methods

This study used a qualitative, individual interview design. Participant recruitment took place at two NHS gynaecology clinics located in the North of England and the Midlands and at one private clinic in the Midlands. Ethical approval was granted by a National Health Service (NHS) Ethics Committee (18/NW/0007) and the Health Research Authority, England. Local Research and Development permissions were granted by agreed NHS trusts.

### Participant inclusion and exclusion criteria

Participants were eligible for inclusion if they were: (1) English speaking women, (2) aged 18 years or over, (3) had previously received a diagnosis of PMDD by a medical specialist and (4) met the clinical criteria for PMDD on the Premenstrual Symptoms Screening Tool (PSST) [25]. Women who did not meet these four basic criteria were excluded.

### Measures

All women were screened using the Premenstrual Symptoms Screening Tool (PSST) [25]. The PSST is a brief 19-item-questionnaire which presents the categorical DSM-IV diagnostic criteria for PMDD as a self-report rating scale. For each item, women are asked to select from four degrees of severity (“Not at all”, “Mild”, “Moderate” and “Severe”) and diagnosis is assessed by comparing each woman’s responses against the DSM-IV criteria [10]. Although assessments of the reliability and validity of the PSST remain to be completed, a study that screened 519 women using this measure identified prevalence rates of PMDD in line with the results of several large prospective studies [25]. For women whose symptoms had been effectively treated, the PSST was completed retrospectively.

### Procedure

A Consultant in Obstetrics and Gynaecology and a Consultant in Sexual and Reproductive Health identified eligible patients on their caseloads and provided them with invitation letters, either by post to their home address or in person during their clinic appointment. These letters outlined the aims of the study and asked women who may be interested in participating to return a completed consent-to-contact form to the research team, in a stamped addressed envelope which was provided. Women who returned this form were then sent information packs by post which included a participant information sheet, a consent form and a PSST questionnaire [25] that they were required to complete and return in a stamped addressed envelope if they wished to take part. Additional women who had heard about the study via word-of-mouth and who had contacted the research team directly by email to express their interest were also sent information packs by post.

Once a signed consent form and completed PSST questionnaire had been received by the research team, the lead author checked that each woman met the inclusion criteria and then contacted them by telephone to arrange a convenient time and location for the interview to take place. During this telephone call, women were given the option of a face-to-face interview at either their home or at the University, or the option of a remote interview by telephone. Women were also asked about their menstrual cycle and whether they still experienced cyclical PMDD symptoms and, for women who confirmed that they did, interviews were scheduled during the follicular menstrual phase in an attempt to minimise participant burden or distress.

All interviews took place during 2018 and were audio-recorded using an encrypted dictation device. At the beginning of each interview, the first author verbally re-consented each participant, in order to ensure that they were still happy to continue, and asked some brief demographic information questions. Participants were then interviewed using a semi-structured interview guide which was developed by the researchers, informed by the aims of the study and the existing literature. Open-ended questions were used to elicit each women’s experiences, views and reflections and all participants were offered the opportunity to raise any additional points. A copy of the interview guide can be found in “Appendix”.

At the end of each interview, participants were given time to reflect on their experience of the interview process and were provided with a debrief information sheet that included contact details for the research team and details of organisations who could offer help and support. In the week that followed their interview, each participant also received a card from the lead author which thanked them for their time and commitment to the study.

The recorded interviews were later transcribed verbatim by both the first author (53%) and an approved and experienced transcriber (47%) and all transcripts were checked for accuracy.

### Data analysis

Data were analysed using Reflexive Thematic Analysis [26, 27]. This method was selected due to its ability to offer rich and detailed insights into a real world problem, alongside its theoretical flexibility when exploring an under-researched population [26]. Our research was underpinned by a critical realist stance, based upon the ontological premise that reality exists, whilst acknowledging that that our understanding of it is influenced by our own perspectives and locatedness [28]. During the analysis the first author used an inductive, data-driven approach, guided by Braun and Clarke’s six key stages [26]. See Table 1 for an outline of this process.

**Table 1:** Analytic stages of the thematic analysis:

Stage of thematic analysis [26]
1. *Familiarisation and immersion in the data:* including transcription of the verbal data, checking transcripts for accuracy and repeated reading of the entire data set looking for patterns and meaning
2. *Generating initial codes:* systematically coding interesting features of the data at the semantic level using a data-driven, line-by-line coding technique. All data was coded at this stage
3. *Searching for themes:* sorting initial semantic codes into broader concepts by collating similar codes together and considering how they may combine to form candidate themes. An excel spreadsheet was used to aid this process
4. *Reviewing themes:* the continued refinement of candidate themes by analysing coded extracts at the latent level, discussions with the research team, examining the fit of themes across the raw data set and using visual aids to develop a thematic map
5. *Defining and naming themes:* identifying the essence of each theme and continuing to analyse data at the latent level, with the aim of generating an underlying ‘story’
6. *Producing the report:* organising and presenting the themes as a coherent narrative, supported by key data extracts

### Reflexivity and rigour

The first author was mindful of both her “insider” and “outsider” positionality and the impact of her multiple roles as a woman and as a healthcare professional upon the questions asked and the findings obtained [29]. This ‘humanness’ transcends all qualitative research and has been identified as an important research tool, if it is appropriately thought about and considered [28]. In accordance with available guidance [30], several techniques were employed to demonstrate rigour during this process: (1) the first author utilised a reflective journal to record methodological decisions, alongside her initial thoughts and impressions of both participant interviews and the transcribed data. In doing so, reflexive thoughts pertaining to the impact, values and assumptions of the first author’s own positionality were acknowledged. (2) Clear audit trails for both coding and theme development were maintained. (3) Themes were reviewed and agreed by members of the research team to consider viewpoints from different perspectives. (4) The adequacy of themes was tested by returning to the raw data set with the initial thematic structure and (5) a thematic diagram was produced in order to make sense of theme connections.

## Results

### Sample

Twenty-two women requested information about the study; twenty returned consent forms and met the inclusion criteria. Three women who returned consent forms did not participate in the study, due to limited time and availability (*n* = 1) or no response to follow up contact from the first author (*n* = 2). The final sample comprised of 17 women. Interviews were conducted face to face (*n* = 15) and by telephone (*n* = 2).

### Sample characteristics

Participants were aged between 20 and 56 years, with a mean average age of 37 years. The median average age at the onset of women’s symptoms was 15 years, ranging from 11 to 38 years and the median average age when women received a diagnosis of PMDD was 35 years, ranging from 20 to 45 years. Women were predominantly white British (*n* = 14; 83%) and had obtained an undergraduate level of education or higher (*n* = 9; 53%). Eight women were married (47%), with the remainder being single (*n* = 8; 47%) and divorced (*n* = 1; 6%) and ten women (59%) reported that they were mothers.

### Findings of the thematic analysis

**Fig. 1:**
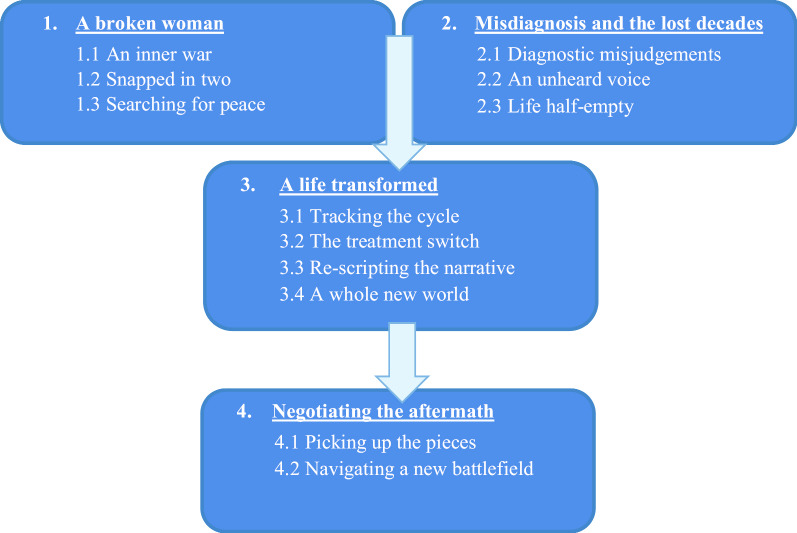
Women’s experiences of receiving a diagnosis of PMDD

The analysis produced 95 initial codes which were grouped into four main themes. Each main theme conceptualises a different aspect of these women’s experiences and contains a number of subthemes. Figure 1 shows a diagrammatical depiction of all main themes and subthemes, illustrating the trajectory and temporal relationship of experiences described by these women who had received a diagnosis of PMDD.

#### Theme 1: A broken woman

The first theme, comprising of three subthemes, outlines the emotional impact of living with PMDD, including the symptoms that the women experienced and their methods of coping.

### An inner war

Women described how for a significant proportion of their lives they had suffered a range of extreme physical and psychological symptoms that they experienced as an inner “battle” against their own body and mind. With symptoms including severe and unexplained period pains and excessive bleeding during menstruation, alongside a variety of emotional changes which were experienced as deeply distressing; women shared how they had often spent many years trying to cope with experiences that they struggled to understand and felt powerless to prevent:

All of a sudden it went pitch black, my emotional mood changed drastically and I could never see any outside things, like things had happened that made me upset or made me dark, so as a very young woman I was wondering why I felt that darkness. I felt like there was no point in living. (Participant 1)

Identifying their psychological symptoms as the most debilitating, women talked about how they had experienced a “rollercoaster” of negative emotions, where their mood would fluctuate dramatically without warning or apparent context:

My mental health was just that bad at the time … it was calming down and then coming again and then calming down and then coming again. (Participant 7)

In particular, women talked about the episodes of deep sadness and despair that they had felt and how these emotions had frequently led to periods of both unexplained and uncontrollable crying:

When I say I feel down; it’s not just feeling down. It’s feeling like your whole body is numb, like I, I’ve had so many times where I’ve felt like if I put my hand on a wall, I’d go straight through it, like that’s the level of sadness. (Participant 3)

In addition to their sadness, women described how their mood would also rapidly swing between other strongly felt emotions, including anxiety and panic attacks which were described as all-consuming and feelings of uncontrollable frustration and anger that the women often recognised as grossly disproportionate to a situation:

It was really feeling just, very low, very hopeless, very tearful, but then, getting very angry as well, so it, it’d swing all over the place. (Participant 9)

### Snapped in two

Using words such as “monster” and “alter ego”, women talked about how the psychological changes that they had experienced often left them feeling like they had transformed into a different person, someone that they did not recognise as themselves:

They [family] would always say to me ‘it’s like you are not yourself’. And I always say that I feel it’s like an alter ego … I am like a completely different person. (Participant 13)

Describing how this transformation would happen very suddenly and with no obvious trigger, women expressed how they were left feeling completely “out of control” and how they were unable to stop themselves from engaging in uncharacteristic and destructive acts:

I couldn’t control the way that I was feeling, I’d cry at the drop of a hat and I’m not particularly a cry, a crying kind of person. It takes quite a lot to get me upset, erm, I just literally could not function. I couldn’t, I didn’t want to get out of bed in the morning, couldn’t sleep at night, erm … just doing stupid things like ripping wallpaper off because I couldn’t cope with the anxiety, the feeling of the anxiety. (Participant 10).

Recalling feeling frightened and fearful about what was happening to them, women talked about how they had tried to hide these changes from those around them. However, by trying to contain and mask their emotions at work and in public, women described how their feelings were often expressed at home, upon loved ones:

No matter how rubbish I was feeling, I could walk into work, put my work face on, get through the shift … inside, all I could think about was wanting to be dead. But, you know, I am at work, I had a duty to other people … At home was different, because I think that when you are around people you love, you can be a bit more free. (Participant 12).

### Searching for peace

Describing how they had felt trapped and alone in their experiences, women talked about the desperation that they had felt to “escape” from their psychological distress; however, each time, they found that there was “nowhere to go”:

It’s like you’re running, you’re trying to run away from yourself, and, but running into a brick wall all the time. (Participant 4)

In response to these feelings, some women talked about relying on the use of drugs and alcohol as a means of escaping from their reality, whilst others described how they had resorted to self-harming behaviours, as a way of trying to manage their emotions. As a result of using these methods, a number of women reported how they developed substantial secondary mental health difficulties, including eating disorders and substance misuse problems:

I drank copious amounts of alcohol, like to the point where I had a problem. That was my coping mechanism, was to just escape. (Participant 3)

Ultimately, women expressed how any relief from their attempts to cope was either temporary or could not be reached, leaving them feeling hopeless and exhausted. From constant thoughts of suicide, through to preparations and in some cases many serious attempts, women talked about the desperation that they had felt to find “a way out” and how taking their own life had often seemed their only option:

I just got to a point where I couldn’t–I didn’t want to live. I just wanted to be dead. (Participant 12)

#### Theme 2: Misdiagnosis and the lost decades

The second theme, comprising of three subthemes, pertains to women’s experiences of seeking help, including the responses that women received from healthcare professionals and the impact of these on their subsequent lives. In particular, this theme captures the many years and life experiences that women lost to their symptoms and highlights the prolonged and enduring nature of their difficulties.

### Diagnostic misjudgements

Over the years of their symptoms, women described how they had been given a variety of diagnoses by healthcare professionals, ranging from anxiety and depression, through to more complex mental health conditions such as bipolar disorder and personality disorders. As a consequence of these diagnoses, women talked about how they had been prescribed a variety of psychiatric medications, often for periods spanning many years:

I had been diagnosed with bipolar disorder, ME, chronic fatigue, depression, and, erm, and given medication for all of those different things (Participant 6)

With no obvious factors to account for the sudden negative shifts in their emotions, women described how they had personally held great doubts about their mental health diagnosis, believing that it did not accurately match their psychological experiences:

I knew it wasn’t that … It was depressing and it was miserable and anxious and everything, but I didn’t feel that it was just that. And they never even did sort of, like, say ‘oh its depression maybe because of this’, they just used to say it’s depression and here’s some pills. (Participant 8)

Struggling to accept the diagnoses that they had been given, some women talked about how they had been reluctant to take the psychiatric medications that they had been prescribed and how they had often felt pressurised into complying with treatments against their better judgement:

I finally agreed to take antidepressants and… [sigh]… against my wish really. But I was willing to try whatever, if they would just listen. (Participant 15)

However, having often adhered to pharmacological recommendations out of desperation, many women reported finding that psychiatric drugs were ineffective in relieving their symptoms and that they added further debilitating side effects to the experiences that the women were already having:

You get to the stage where you are on so many antidepressants that you sort of… you can hear your own voice slurring and everything else and you’re still thinking inside ‘but I’m still not right… something is still completely wrong’. (Participant 2)

### An unheard voice

Concluding that their mental health diagnosis was inaccurate, women talked about how they had believed that there must be another explanation for their psychological difficulties. However, when they raised these concerns, women described how they were met with an unwillingness from healthcare professionals to consider, or explore, any possible alternative causes.

The other GP, he just kept knocking me back time and time again—you are depressed, you’re depressed, you’re depressed. (Participant 5)

With no visible reasons for their distress, women talked about how they had felt that were being appraised negatively by healthcare professionals, including General Practitioners (GPs) and Accident and Emergency staff, as “faking” or exaggerating the severity of their symptoms and described how this led to scenarios in which they had felt ridiculed and belittled when they had tried to assert their opinions:

I just felt like everybody [healthcare professionals] thought that I was making it up. And there is nothing worse is there than when you feel that everyone thinks you are lying. (Participant 15)

From feeling repeatedly “dismissed” with antidepressant medications, to being told that their mood changes were simply part of a “normal” female experience, women described how over the years that followed, their pleas for recognition went both unacknowledged and unheard. Using words like “desperate” and “crying out for help”, women talked about their feelings of helplessness and hopelessness as *“every single door was just being closed*” to them (*Participant 2*):

I said I can’t carry on like this, I just can’t carry on like this-I need help. I was crying out for years and years and years. And there was no one listening. (Participant 11)

In their quest to find answers, women reflected upon how they had needed to fight multiple battles, both against their symptoms and against the professionals from whom they had sought help:

You’re fighting everything and everyone, including yourself. You don’t have the energy, you know, to deal with everyone else … I was coming across as a hysterical young lady and I think really big assumptions were made, and I just felt that my opinion and voice counted for nothing. (Participant 15)

### Life half empty

With the onset of symptoms typically beginning during adolescence, women described how their life had stopped at a time when new opportunities should have been opening up to them and how, as a result, they felt that they had lost many of the things that were important to them: “I had so many hopes for my life that I’m never going to be able to do because of this condition, like I did so well at school and I thought I’d have a really good degree, and I wanted to do a Masters, erm, I wanted to help other people. But I don’t feel like I’ve been able to do any of that because of this condition, erm, honestly, I think it’s… it’s… it’s broken me, completely.” (Participant 3)

When describing the limitations that their symptoms had placed upon their education and career opportunities, women talked how they had struggled to concentrate during their school and college years and how they had often ended up withdrawing from university or higher education. With regards to work, women talked about how their fluctuating psychological symptoms had often meant that they struggled to meet the demands of their job and how they had regularly terminated employment due to feeling unable to cope:

You end up crying, you end up at work with your face crying, you are emotionally exhausted and you are expected to go in and do a full days’ work and be this happy jolly camper when deep down you want to be somewhere else, and some days you don’t want to be there at all. (Participant 14)

In their personal lives, women described the negative impact that their symptoms had on their relationships with others. From feeling rejected by those around them, to isolating themselves from loved ones in a bid to protect them; women recalled the breakdown of their romantic relationships and the disappearance of their longstanding friendships. For women who were mothers, they talked about having felt unable to care for their children and their deep regret for not having been able to be the parent that they wished to have been. In particular, women talked about the feelings of immense loss and isolation that they had felt, resulting from, alongside descriptions of damaged family relationships and the despair and helplessness of those who tried to remain supportive:

You know work wise I just held onto a job, the happiness of being with my children dissolved into nothing really and my relationship really suffered with my husband because of it. So it’s very hard.” (Participant 5)

#### Theme 3: A life transformed

The third theme comprises of four subthemes and captures women’s experiences of recognising PMDD and the impact that this diagnosis had for their self-identity, subsequent treatment and lives.

### Tracking the cycle

Women reported how over time both they and those close to them had observed how their symptoms recurrently peaked and abated, for some, resulting in monthly admissions to the emergency department each time they reached crisis point. In addition to this, a number of women reported noticing how their symptoms had been absent during the nine months of their pregnancy and how they had returned after birth:

The only time that I have been stable was when I was pregnant and breastfeeding. My mood was stable, I was stable, I didn’t get nothing because you don’t get no periods and even my husband would say the most stable he has known me was when I was pregnant. Other than that he has just felt like he has lived with a whirlwind. (Participant 7)

By using diaries to track their experiences over several months, women described how they had been astonished to find clear cyclical patterns, during which their negative feelings dramatically increased in severity during the weeks preceding menstruation, followed by a sense of great relief when their period arrived:

Keeping a diary is an essential beginning to this—to actually understand what is going on…that’s the key to it. Understanding the ups and downs of each month and being able to work out that there is a pattern. (Participant 5)

For some women there appeared a clear delineation between two “bad” weeks in the lead up to menstruation, followed by two “good” weeks, whereas other women reported experiencing only a few days of normality following menstruation, before their cycle and psychological symptoms recommenced:

Some months I would only have a matter of a couple of days where I actually felt okay. (Participant 10)

This new evidence for cyclical connection was identified as a pivotal step by women and the beginning of a renewed determination to seek answers. Women described conducting vast amounts of research via the internet and presenting this to healthcare professionals, as well as proactively seeking out hormone specialists and paying privately for help:

I went home and I started researching it, started watching YouTube videos, started reading papers, everything like that. And it was like the more I was reading, the more I was like right okay, I know that I have got to sort this. (Participant 13)

Finally, women talked about the moment that they discovered PMDD and their feelings of immense relief at the realisation that their experiences with PMDD-related symptoms suddenly made sense. In contrast to their previous mental health misdiagnoses, women expressed how PMDD finally fitted with their experiences, accurately capturing the recurring psychological symptoms that they had been unable to fathom for so long:

It made everything make sense and everything just clicked into place—it really did make sense that actually I wasn’t mad, like I thought I was going mad, and I drove myself mad. (Participant 7)

### The treatment switch

All of the women perceived the treatment that they had received for PMDD as both life-changing, and life-saving. After being given a GnRH agonist drug to eliminate the hormonal shifts that had been making them so unwell, women talked about feeling as though a “switch” within them had been flicked and how this treatment had provided the ultimate evidence that their difficulties were related to their menstrual cycle:

I felt like my whole world had turned upside down again- I felt amazing—it was like someone had switched the light from on to off. It really was that severe, that quick. (Participant 15)

For some women, the realisation of how it felt to be calm and unburdened by their mental health symptoms was described as overwhelming, with one participant expressing how until she had received treatment, she *“never actually knew what it was like to be happy.” *(*Participant 3*). For others, they described the disbelief that they had felt at the sudden disappearance of the psychological symptoms that they had been living with for so long:

I had never felt that well in my life since I was eleven, I couldn’t remember anything before I was eleven really. All I know of the last sixteen years was self-destruction and anxiety and eating disorders and basically just surviving and getting through each day and hoping for the best, and, erm, I just kept saying to my mum, like, this can’t be real, is this how people actually feel? (Participant 17)

### Re-scripting the narrative

The revelation that their experiences were caused by an undiagnosed medical condition appeared to have a significant impact on each woman’s understanding of her own identity. Prior to their diagnosis, women described finding it difficult to understand their own experiences:

I couldn’t understand why I felt like that, because I had parents that had always been there for me, and I had a good upbringing, so it wasn’t that [crying]. It was just this terrible darkness that hit me on and off, on and off, on and off. (Participant 1)

With no identifiable cause or trigger for their feelings, some women talked about fearing that they really were seriously mentally unwell or “crazy”, whilst others described concluding that their symptoms must be a fundamental flaw in their personality. The feelings of shame and self-hate that many of the women had experienced due to being unable to overcome their mental health difficulties resulted in an extremely damaged sense of self: women described feeling “worthless and “bad”, how they had blamed themselves for the situation that they were in:

I just thought that I was a bad person. And that was that—I just thought that I was horrible and that’s the way that I was. (Participant 12)

However, the new understanding that their symptoms had a physical cause appeared to result in a transformed sense of identity: women described how they were suddenly able to make sense of their past experiences and how this knowledge had helped them to understand how the things that had happened were beyond their control:

I also feel like now I know why, like I know why I feel so anxious sometimes and why I feel so sad. I know it’s not my fault, which is probably the main thing, I know it’s not my fault now, I’m not just a bad person. (Participant 3)

By being able to separate who they were from their symptoms, women talked about feelings of increased confidence and self-worth, alongside the realisation of how “strong” they had actually been in coping with their emotional difficulties for so long:

I realise now, I’ve been carrying this for so many years myself [crying] and I realise I am not weak, I am strong. I’m very strong. (Participant 1)

### A whole new world

With a new diagnosis opening up new avenues of support, women talked about the overwhelming feeling of relief that they had felt to reach a specialist in PMDD, where the severity of their symptoms was finally taken seriously. In contrast to feeling dismissed, women described facing a professional who showed a genuine interest in their experiences and who wanted to hear what they had to say:

He [Specialist] was the first person that actually took my symptoms seriously, because when I told him what I had been feeling, the first thing he said is that must be awful for you. And nobody, in 17 years, family, friends, nothing, had ever said that to me. (Participant 12)

From receiving validation that they were battling a real condition, to making and embarking upon treatment plans, women described the sense of safety that they had felt in being treated by someone who understood and how incredible it had felt to feel truly supported for the first time:

I think to actually know that it has been recognised, that somebody is there supporting you, I think is… it was, it is completely brilliant. And even after having the diagnosis I rang the [Specialist’s] secretary a few times, bawling hysterically going ‘I really need to see somebody again’, and it was just the support- and I think that they already knew that they were dealing with people who were having these issues … patients that are completely, you know, lost really. So I think that dealing with somebody that understood, that knew, and I remember leaving that appointment thinking ‘oh my gosh—I actually have this. (Participant 2).

After obtaining effective treatment for their condition, women described similar transformations in both the quality and enjoyment of their lives. From promotions at work to embarking upon entirely new careers, women described beginning to live the life that they had once envisaged:

When I’m on [chemical menopause] I’m fine, like it’s amazing [laughs]. Yeah, like I’ve been able to keep a job, I’m in a relationship, I’m able to actually think about my future. (Participant 3)

Having felt isolated for so long, women also identified their joy at being able to reconnect with others and how, as a result, their family and romantic relationships had both strengthened and blossomed:

He felt he got his wife back, and I got my life back. You know it’s very true actually—it really does summarise how the treatment has actually completely changed our lives. (Participant 5)

#### Theme 4: Negotiating the aftermath

The final theme, comprising two subthemes, relates to the residual difficulties that followed women’s experiences of misdiagnosis and the new challenges that a life with PMDD presented.

### Picking up the pieces

Whilst diagnosis and treatment were described as transformative, for many of the women interviewed, their illness had impacted heavily upon both their adolescent and adult lives. When reflecting on this, women often talked about their sadness in relation to their past experiences and the struggles that they had in coming to terms with what had happened to them:

[Crying] You can feel sad looking back, obviously that’s probably why I’m crying; it’s so much suffering, emotionally suffering. (Participant 1)

The differences between their expected and actual life experiences were extremely hard for some women to bear and, with no specialist emotional support available to them, women described how they were left grieving for what might have been, had they received a correct diagnosis sooner:

I feel like I have wasted my life, erm … had all those opportunities… to be something. And I have got the intelligence, but I haven’t got a direction [crying] … that’s how it feels. (Participant 8)

In addition to their grief, women described the challenges of needing to reinvent and redefine themselves, including re-learning who they were now and how they reacted to things, after having felt “lost” for so long:

I have come out the other end of it, as in I have got this, and everything is going to get better and is 100 times better… but who the hell am I now?… Who am I and what do I do? (Participant 8)

Women also talked about how finding a new sense of normality was difficult for those around them, with families and loved ones also needing to relearn who the woman was without her symptoms and needing to be able to adjust to this sudden change following treatment:

It does still affect us today—even though I am fine now, erm… and I guess mainly because my husband doesn’t know how to act around me anymore because he has been treading on eggshells for so, so long now that now maybe he won’t tell me things or he’ll ring me up from work and say ‘oh I’m stuck in work, are you going to cope? … he doesn’t know how to react still, which is hard when I think having a hysterectomy for me literally flicked that switch.” (Participant 2)

From an ongoing mistrust of medical professionals, to carrying remaining guilt for the ways in which they had previously treated loved ones, women described how their experiences had lasting consequences. However, for women further into their journey towards self-recovery it seemed that there was a place of acceptance that could be reached, where they were able to place a greater emphasis on what they had, rather than on what they had lost:

Talking about it made me realise how much this has affected my life. Like, how, awful it’s been and how tiring and how desperate I’ve been at times, and that, I’m so grateful to even be here now. (Participant 3)

### Navigating a new battlefield

Following their diagnosis, women also talked about needing to negotiate the new challenges that a life with PMDD presented. Despite having a clinical diagnosis of PMDD, given by a specialist in the field, many women described a continued battle for recognition and care from healthcare professionals who refused to acknowledge or discuss their condition as a legitimate illness:

My mental health team were still saying like we don’t really like believe in this kind of thing, like, never really heard of it. (Participant 17)

Finding that there was a significant lack of awareness of PMDD in the medical community, women also talked about their frustrations at continuing to feel misunderstood and stigmatised, particularly by male medical doctors who they often identified as dismissive of menstrual-related concerns:

The [male] doctor that I saw told me that there was no such thing as PMDD, there was no such thing as PMS and this it is just something that women say. (Participant 13)

In addition, women described the complexities of trying to obtain treatment for a condition for which there are, to date, no approved disorder-specific medications and how this created further barriers to their care. From experiencing a variety of negative side effects from ovulation suppressing drugs, to finding that GnRH agonist treatments eventually stopped working, women talked about needing to trial a number of different medications before eventually finding that they had reached the limit of the pharmacological options available to them:

You can tell when it gets to three months, I just drop, like I’ve had my [GnRH] injection this week, but last week I was just frozen to my bed, er, I couldn’t, I didn’t go to work. (Participant 3)

With total hysterectomy combined with bilateral oophorectomy considered a last resort treatment when the medical management of PMDD has failed [22], both women yet to have children and women who had been hoping to add to their families talked about the difficulties of having to make a choice between their own life and carrying a future biological child. Fears about how a pregnancy could be achieved without becoming acutely unwell resulted in some women describing how they had made a conscious and self-acceptable decision not to become mothers, whereas for others, they described how facing a lifetime of infertility remained incredibly painful, adding a further dimension of grief to the life experiences that they felt that they had lost:

Not being able to have children, that’s a great sadness. Erm, yeah because, you see, you know, I, I could barely look after myself, so I couldn’t, even think about bringing a child, you know, into the world, because I just wasn’t emotionally stable enough. So, erm, so, yeah, I couldn’t have children. So that’s a great sadness, that’s a big impact. (Participant 6)

## Discussion

The aim of this qualitative study was to explore women’s lived experiences of both having PMDD and of receiving this diagnosis. Consistent with what is currently known [1–5, 8], the themes subsequently generated highlight the severe psychological changes that women with PMDD experienced prior to the onset of menstruation and stress the significant and debilitating impact of these symptoms for the women’s subsequent quality of life. In addition, this study identified the challenges that this sample of women experienced in their attempts to be heard by healthcare professionals and how this often resulted in psychiatric misdiagnoses and an average delay of 20 years before PMDD was correctly identified and treated. Although treatment for PMDD was described as transformative, important narratives pertaining to the residual difficulties that stemmed from many years of misattributed symptoms were generated, alongside the challenges that a new life with PMDD presented.

Whilst psychiatric misdiagnoses have previously been reported for women with PMDD [3], the findings of this study serve to further evidence the vulnerability of this population to serious clinical misjudgements and highlight the challenge for healthcare professionals of distinguishing between PMDD and other serious psychiatric disorders. With the psychological symptoms of PMDD presenting as similar to mental health conditions, identifying the cyclicity of women’s symptoms was key to achieving an accurate diagnosis in this study. When left undiagnosed and untreated, our findings showed how women with PMDD developed substantial secondary difficulties in their attempts to cope, including eating disorders, substance misuse problems and serious suicidal behaviours which are likely to further complicate a clinical picture. Therefore, in order to prevent women with PMDD from experiencing prolonged periods of unnecessary psychological distress, accurate and timely diagnosis of this condition is paramount.

In order to further understand the challenges that the women in this study experienced in their attempts to be heard, including their descriptions of how their concerns were repeatedly dismissed and rejected, it is important to consider the wider literature relating to the stigmas that are identified to exist in healthcare settings. It is a well-documented finding that individuals who are viewed as ‘psychiatric patients’ are frequently identified to experience a lack of respect in medical settings [31], with evidence that healthcare professionals often fail to act on a patient’s concerns when mental health labels are present [32]. By presenting with mental health symptoms and being erroneously diagnosed with serious psychiatric disorders, our results suggest that the women in this study become direct recipients for these negative attitudes, limiting any further exploration of their difficulties and resulting in professionals missing critical opportunities to correctly identify PMDD.

In addition to the stigma attached to mental health, women’s descriptions of feeling that they were being viewed as “lying” about their experiences, alongside their accounts of being told by professionals that PMDD was not a real condition, may also be indicative of wider biases that exist for women within the healthcare system. There is a growing body of evidence to substantiate a significant gender gap in the treatment of health concerns, in which women’s experiences are less likely to be taken seriously by medical professionals [33], together with a greatly increased vulnerability for psychogenic misdiagnosis [34, 35]. Importantly, this bias appears amplified when it comes to reproductive health: there is evidence to show that women who present with menstruation-related concerns are repeatedly dismissed and ignored by medical professionals [36, 37], as well as views that their symptoms are “exaggerated or imagined” (p. 207) [37]. Therefore, the findings of this study, along with the wider literature, indicate that women with PMDD may be prevented from reaching an accurate diagnosis by the stigmas that are commonly attached to both mental and women’s health conditions.

Following receiving a correct diagnosis, the emotional task for women of reconstructing both their life and identity, alongside managing their grief for lost decades and needing to reach a place of acceptance, were identified as profound in this study and should not be underestimated. Identity changes have frequently been documented following the onset of mental and physical illnesses, describing how an individual’s sense of self can become lost or disrupted [38–40]. However, with the onset of PMDD typically commencing during adolescence, at a time believed critical for identity formation [41], our findings indicated that the women in this study had often incorporated their symptoms into their self-concept from a young age and how, following receiving an accurate diagnosis and treatment, these women were faced with the sudden task of reconceptualising both their life and who they were as a person. In order to help women to manage this adjustment and to process their experiences, access to psychological support is recommended.

### Future directions

The experiences of the women detailed in this study provide a stark illustration of the real world consequences of misdiagnosed PMDD and highlight how this condition is in desperate need of wider focus and attention moving forward. Increasing the awareness and understanding of PMDD in the medical community appears paramount, specifically amongst front line professionals, such as General Practitioners, as well as within mental health and psychiatry teams to which women are likely to present. In addition to this, more research is needed to develop and evaluate PMDD screening measures, alongside training for clinicians on how to assess women with possible symptoms. Encouraging all women with mental health difficulties to assess their own menstrual cycle and symptoms using diaries or online symptom tracker apps may also be beneficial in facilitating earlier diagnosis. However, more research is required to assess if this is feasible and acceptable for women, and to further determine if diaries and the tracking of cycles lead to earlier detection and treatment.

### Strengths and limitations

The themes identified in this study provide rich and detailed insights into women’s experiences of both having and receiving a diagnosis of PMDD. However, these findings should be considered in the context of some methodological limitations. Firstly, although the sample achieved was heterogeneous in terms of age and location, in which women were successfully recruited from across the United Kingdom, the majority of women in this study were white British (83%) leading to a possible cultural bias. Furthermore, this study only included women who had received an affirmative diagnosis of PMDD and, by the nature of the recruitment method, were subsequently under the care of an experienced medical specialist. Whilst this was viewed as a strength of the study, because this recruitment method enabled the authors to ensure that the women had an accurate diagnosis of PMDD, in comparison to previous studies in which diagnoses are identified as ‘provisional’ [42, 43], it is acknowledged that the participants in this study represent a subset of women who had been successful in receiving help.

Likewise, variability is identified to exist in the level of symptom burden of PMDD, with some women being more severely afflicted than others [1]. It is therefore important to acknowledge that the sample of women in this study were particularly afflicted and that the severity of their experiences do not necessarily represent all women with PMDD. In order to address these limitations, it will be important for future research to also examine the experiences of women who are recruited via different sampling methods and who have different backgrounds to the current sample in order to enable wider conclusions to be drawn.

## Conclusions

It is vitally important that healthcare professionals are able to accurately distinguish the difference between PMDD and severe psychiatric disorders to ensure that women with this condition receive appropriate and timely support. In order to achieve this, identifying the cyclicity of women’s symptoms is key. Future directions include the need for a greater understanding and awareness of PMDD within medical communities, alongside training for healthcare practitioners in PMDD assessment. Women with mental health difficulties should be encouraged to assess their own symptoms, using diaries or an online symptom tracker app.

## Data Availability

The data generated during this study consists of transcriptions of the audio-recorded interviews. Most of the relevant data are presented as quotes in the text. We do not wish to share the full text transcripts at present because EO and AW are considering a further analysis of the data with a different research question at present.

## Appendix

### Interview guide

1. Individual experience of PMDD
  - Tell me about your experience of having PMDD
    Probe for:
    - Symptoms (*what did/do you experience?*)
    - Onset (*can you tell me about when these experiences began?*)
    - Impact on life domains e.g. education, career, family relationships, mood, sense of self (*how did these experiences impact on the different areas of your life? Was there any impact on your career, education, relationships, mood, sense of self?*)
    - Severity e.g. worst points (*what would you describe as the worst points of your experiences?*)
2. Experiences of receiving a diagnosis of PMDD
  - Tell me about your journey to receiving a diagnosis of PMDD
    Probe for:
    - Timelines—length of time from onset to diagnosis (*How long do you think it took from first noticing symptoms, to receiving a diagnosis?*)
    - *Who was first to identify that you may have PMDD?* (individual sufferer or medical professional)
    - Who made diagnosis (e.g., gynaecologist, psychiatrist, GP, etc.) (*Who did you approach for help/seek advice from about your experiences?*)
    - Process of obtaining diagnosis—e.g. NHS, privately. (*Did you seek medical support from the NHS or privately? What were the reasons for this?*).
    - Any misdiagnoses before PMDD (*Were you diagnosed with, or treated for any other conditions, before PMDD was identified?*)
  - What did you find helpful in your journey to diagnosis?
    Probe for:
    - Any specific tracking methods used (*did you use any ways of measuring or tracking your experiences?*)
    - Type of health professionals consulted (*did you consult with different health professionals?*)
    - Own awareness of condition (*Were you aware that you might have PMDD, before your diagnosis? What made you think this?*)
  - What did you find unhelpful in your journey to diagnosis?
    Probe for:
    - Any barriers that participants faced to diagnosis e.g. others views regarding PMDD; awareness and receptiveness of health professionals; previous mental health labels
    - Were there were any barriers to getting your diagnosis? What were these?
3. Consequences of diagnosis
  - Were there any consequences of receiving a diagnosis of PMDD? (Positive or negative).
    Probe for:
    - What was it like for you to receive this diagnosis? What was it like for those around you?
    - Are there any positives/negatives of PMDD diagnosis?
    - Has your life changed in any way since receiving a diagnosis of PMDD?
    - Views—Did this diagnosis change how you viewed yourself? Did it change how others viewed you?
4. Advice for others
  - *From your experiences, what advice would you give to other women who may have PMDD?*
    Probe for:
    - What do you know now, that may have been useful to have known earlier?
    - Is there anything that you did, or that others did, that was important in obtaining your diagnosis of PMDD?
    - In retrospect, is there anything that would have improved your path to diagnosis?
    - What advice would you give to health care professionals around PMDD diagnosis?
      Probe for:
      - What did professionals need to know in order to have helped you sooner?
      - Is there anything that they could have done to improve your experiences of diagnosis?
5. Summary
  - Do you have any further points that you would like to make?
  - Is there anything that I have not asked you, which you feel is important in your experience of PMDD diagnosis?
  - Do you have any questions?

## Abbreviations

DSM: Diagnostic and statistical manual of mental disorders
GnRH: Gonadotropin-releasing hormone
GP: General practitioner
ICD: International classification of diseases
NHS: National Health Service
PMDD: Premenstrual dysphoric disorder
PMS: Premenstrual syndrome
PSST: Premenstrual symptoms screening tool
